# Comparisons of Cytokines, Growth Factors and Clinical Efficacy between Platelet-Rich Plasma and Autologous Conditioned Serum for Knee Osteoarthritis Management

**DOI:** 10.3390/biom13030555

**Published:** 2023-03-17

**Authors:** Pen-Gang Cheng, Kuender D. Yang, Liang-Gie Huang, Chi-Hui Wang, Wang-Sheng Ko

**Affiliations:** 1 Department of Orthopaedics, Fu-Ya Medical Clinic, Taichung 40764, Taiwan; 2 Department of Medical Research, Mackay Children’s Hospital, Taipei 10449, Taiwan; 3 Department of Medical Research, Mackay Memorial Hospital, Taipei 10449, Taiwan; 4 Department of Stomatology, Taichung Veterans General Hospital, Taichung 40705, Taiwan; 5 Department of Orthopaedics, Cheng-Ching General Hospital, Taichung 40764, Taiwan; 6 Department of Internal Medicine, Kuang-Tien General Hospital, Taichung 43302, Taiwan

**Keywords:** autologous conditioned serum, platelet-rich plasma, osteoarthritis, knee, intra-articular injections

## Abstract

This study aimed to directly compare the contents and the clinical efficacy of the two autologous blood-derived products, platelet-rich plasma (PRP) and autologous conditioned serum (ACS) for osteoarthritis (OA) treatment. The contents of standard-prepared PRP and ACS prepared at 37 °C for 1 h, 3 h, 6 h, and 24 h from healthy volunteers were compared. The clinical efficacy of pain relief in patients with Stage III knee OA was evaluated by a patient-reported visual analog scale (VAS) pain rating. PDGF-BB levels in ACS 1 h were significantly higher than those in PRP, and the levels in ACS preparations remained stable. IGF-1 level of ACS 24 h showed a significant increase compared to those of other ACS preparations and PRP. ACS 3 h showed a turning of IL-1Ra level and revealed a time-dependent increase up to 24 h. ACS 6 h showed a turning increase in TNF-α levels. ACS 3 h was chosen for clinical comparison with PRP. The reduction in pain VAS in the ACS group was significantly more compared to those of the PRP group (*p* = 0.028). However, PRP showed significant earlier improvement (*p* < 0.001). Conclusion: ACS contained higher levels of PDGF-BB and IL-1Ra and provided better improvement in pain relief compared to PRP.

## 1. Introduction

Osteoarthritis (OA) is one of the most common chronic and degenerative diseases in the aged population over 60 years, and knee OA is the major type [1]. The key symptoms are joint pain, swelling, and stiffness, which can interfere with the patient’s ability to keep normal daily activities, and further worsen their quality of life, resulting in disability and a heavy health burden worldwide [2]. Furthermore, knee OA has been observed that related to an increased risk of all-cause mortality in the elderly [3]. Currently, both non-pharmacological and pharmacological approaches for OA management are used in clinical practice, including weight loss, supervised excise, and neuromuscular training for lifestyle modification, and topical, oral, and intra-articular injection of effective medications [4]. However, the pathogenesis of OA remains uncertain, accompanied by no curable treatment for OA yet. Most interventions aim to manage pain to reduce the physical and psychological stresses of patients since pain closely relates to a reduction in quality of life and ability of daily activity [5]. 

Accumulated evidence indicates that inflammation responds to the symptomatic features of knee OA, including pain, articular cartilage degradation and mild synovitis [6,7]. Infiltration of inflammatory cells in the synovium is characterized as synovitis and observed by imaging and histology [8]; the presence of synovitis is related to worse joint symptoms and cartilage degradation [9,10,11]. Researchers have noticed that patients with OA had higher levels of pro-inflammatory cytokines, including tumor necrosis factor (TNF) and interleukin (IL)-1, which play an important role in the development of OA [12,13]. Suppression of inflammation in the knee can increase mobility by slowing down the symptoms including pain [14]. 

Autologous blood-derived products have gained attention for OA treatment in recent decades [15,16]. Platelet-rich plasma (PRP) is the first one used in the clinic since the 1950s for OA treatment; the favorable outcome has been demonstrated in relieving pain or at least reducing pain intensity, and improving function in patients with knee OA before arthroplasty is absolutely required [4,17]. Previous studies have suggested that intra-articular injection of PRP has anti-inflammation effects on the healing of cartilage, pain relief, and reduction in the severity of synovitis [18,19,20]. The anti-inflammation effects of PRP are not only from concentrated platelets but also cytokines, chemokines and growth factors released by activated platelets for OA treatment [21,22]. Many studies have supported the use of PRP in knee OA; however, controversial results also exist [23,24,25,26]. The sufficiency of the evidence is doubted due to the heterogeneity of the results from the mixed stage of OA in the studied population, the injection frequency, contents and amount of PRP, and the preparation protocols of these products [4]. 

Autologous conditioned serum (ACS) is another product prepared from the patient’s own blood, composed of enriched cytokines, chemokines, and growth factors that are secreted by platelets and blood cells after stimulation with glass beads [27]. Previous studies have supported the therapeutic effect of ACS for OA; the anti-inflammatory properties of ACS are mainly mediated by the function of IL-1 receptor antagonist (IL-1Ra) to inhibit IL-1 in the OA treatment [27,28,29,30,31]. The first device developed for processing ACS was originally branded as “Orthokine” in the late 1990s. The investigators found that incubation of blood with glass beads for 24 h at 37 °C could stimulate the rapid production of IL-1Ra from macrophages, monocytes, and also platelets [28,32].

In this study, we attempted to optimize the ACS treatment for knee OA. ACS was prepared by a product named PRPII sterile glass beads containing a tube in a series of incubation time points. These preparations were compared to the standard preparation of PRP regarding the contents of cytokines and growth factors, including tumor necrosis factor (TNF)-α, fibroblast growth factor (FGF)-1, IL-1Ra, platelet-derived growth factor (PDGF)-BB, and insulin-like growth factor (IGF)-1. Clinical efficacy on pain relief was evaluated by a visual analog scale (VAS) of pain rating in patients with knee OA. 

## 2. Methods

### 2.1. Participants

For comparing the contents of PRP and ACS, blood samples collected from 12 healthy volunteers were used. The clinical efficacy of PRP and ACS was evaluated in patients with Kellgren–Lawrence Stage III knee OA. Patients who were admitted to our institute due to unilateral or bilateral Stage III knee OA diagnosed by certified physicians were asked for consent to participate in this study. Patients aged <18 years, having major neurological diseases, active thrombovascular diseases, or joint infections were excluded. As an open-labeled study, patients could select to receive either PRP or ACS treatment after being informed of the complete steps of PRP or ACS treatment, including the preparation methods, treatment process and efficacy evaluation by the investigators. The possible benefits and disadvantages were also explained. Patients were treated with 3.5 mL of either PRP or ACS fresh prepared for each treatment appointment every 2 weeks five times. The procedure was modified according to previous studies and our clinical experience [27,33,34,35]. Clinical efficacy on pain relief was evaluated by VAS of pain rating. The study protocol was approved by the Institutional Review Board of Kuang-Tien General Hospital (KTGH 11105). All participants provided signed informed consent.

### 2.2. PRP and ACS Preparation for Cytokine Measurement

PRP was prepared from 10 mL of blood from each participant, 0.5 mL sodium citrate was added as the anticoagulant and immediately centrifugated at 2700 rpm for 12 min, and 4 mL of PRP was isolated. Platelets in PRP were activated by adding 0.45 mL of 10% calcium gluconate. ACS was prepared from 10 mL of blood from each participant. Blood samples were collected and transferred into the PRPII sterile glass beads containing tube (Pen-Ling Biotechnology Co., Ltd., Taichung, Taiwan), whose diameter is 16.2 mm and the length is 129.2 mm, with one air pore on the top and medical grade glass beads inside, and incubated at 37 °C for 1 h, 3 h, 6 h, or 24 h before centrifugation at 4000 rpm for 5 min. ACS was harvested using a spinal needle through the air pore. Both PRP and ACS were prepared using non-fasting blood samples. Cytokines and growth factors including TNF-α, FGF-1, IL-1 Ra, PDGF-BB, and IGF-1 were measured using enzyme immune assay kits according to the manufacturer’s instructions (Blossom Biotechnologies, Inc., Taiwan).

### 2.3. Statistical Analyses

For sample size calculation, 30 pairs of participants were set for a power of 0.8, an alpha level of 0.05, and an effect size of 0.5 on the IL-1Ra and PDGF-BB levels. Cytokine levels were presented by the median and interquartile range (IQR), and the comparisons among the four preparation conditions of ACS and PRP were accessed by a nonparametric Friedman’s test. If the result of the Friedman’s test obtained statistical significance, a post-hoc Wilcoxon Signed Rank test was performed to compare every two conditions. Bonferroni correction was applied for the above post-hoc pairwise comparisons. For the demographic and clinical data from the 60 patients with knee OA, categorical variables were expressed by count and percentage, and continuous or ordinal variables such as age, body mass index (BMI), pain duration, pain VAS, and change in pain VAS from baseline were presented by median and IQR. The associations among the categorical variables versus treatments (PRP and ACS) were tested with Fisher’s exact test. Moreover, the differences between treatment groups for the continuous or ordinal variables were tested with the non-parametric Mann–Whitney *U*-test. Two-sided *p*-values less than 0.05 would be considered statistically significant. All statistical analyses were accessed by the software IBM SPSS Statistics 25.0 (IBM Corp., Armonk, NY, USA).

## 3. Results

### 3.1. Comparisons of the Contents between PRP and ACS

#### 3.1.1. FGF-1 Levels

**Figure 1:**
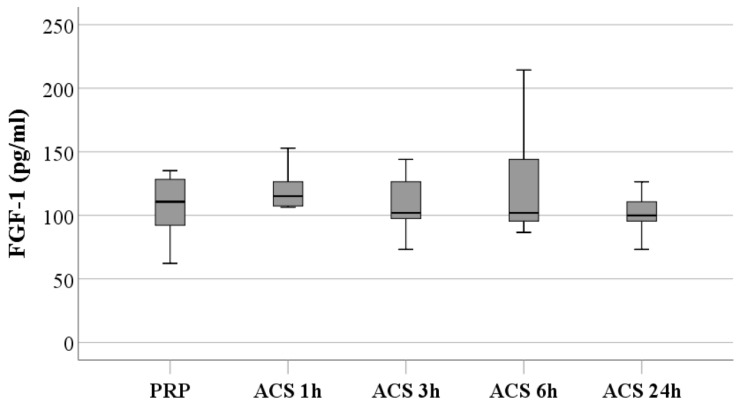
Fibroblast growth factor (FGF)-1 levels of platelet-rich plasma (PRP) and autologous conditioned serum (ACS) with different incubation times. No significant difference was found.

The median FGF-1 level of PRP was 110.7 pg/mL (95% CI, 92.2 to 128.3 pg/mL), and the median FGF-1 levels of ACS preparations for 1 h, 3 h, 6 h, and 24 h were 115.1, 101.9, 101.9, 99.9 pg/mL, respectively. It is maintained stable during the 24 h of incubation (Figure 1 and Supplementary Table S1). There was no significant difference in FGF-1 levels between PRP and ACS preparations. 

#### 3.1.2. PDGF-BB Levels

**Figure 2:**
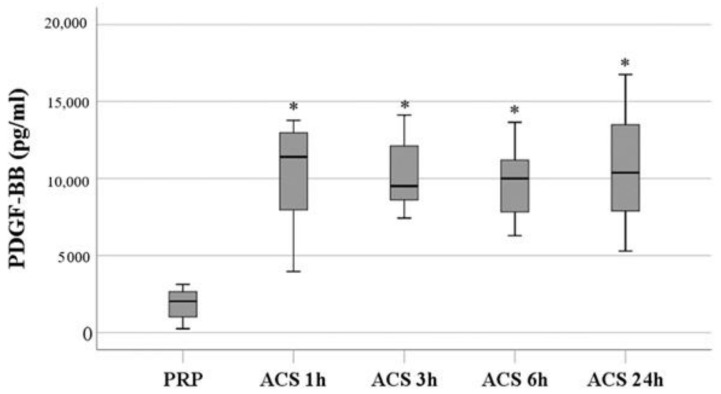
Platelet-derived growth factor (PDGF)-BB levels of platelet-rich plasma (PRP) and autologous conditioned serum (ACS) with different incubation times. * indicates a significant difference as compared to PRP.

PDGF-BB levels of ACS preparations for 1 h, 3 h, 6 h, and 24 h were significantly higher than those of PRP, with the medians of 11,414.8, 9512.7, 10,001.2, 10,382.5 vs. 2035.2 pg/mL (*p* < 0.0005). However, there was no significant difference within the ACS preparations (Figure 2 and Supplementary Table S1).

#### 3.1.3. IGF-1 Levels

**Figure 3:**
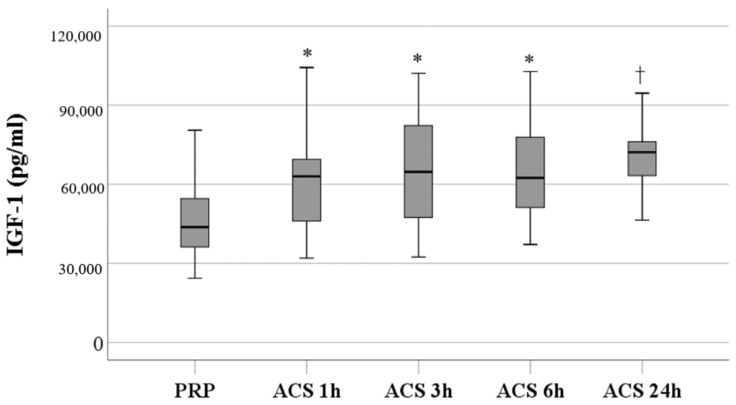
Insulin-like growth factor (IGF)-1 levels of platelet-rich plasma (PRP) and autologous conditioned serum (ACS) with different incubation times. * indicates a significant difference as compared to PRP; † indicates a significant difference as compared to PRP and ACS 1 h.

IGF-1 levels of ACS preparations for 1 h, 3 h, 6 h, and 24 h were significantly higher than those of PRP, with the medians of 63,019.8, 64,724.5, 62,477.9, 72,194.0 vs. 43,790.8 pg/mL (*p* < 0.0005). IGF-1 level of the ACS preparation for 24 h was significantly higher than that of the ACS preparation for 1 h, with a median of 72,194.0 vs. 63,019.8 pg/mL (*p* < 0.0005) (Figure 3 and Supplementary Table S1).

#### 3.1.4. IL-1Ra Levels

**Figure 4:**
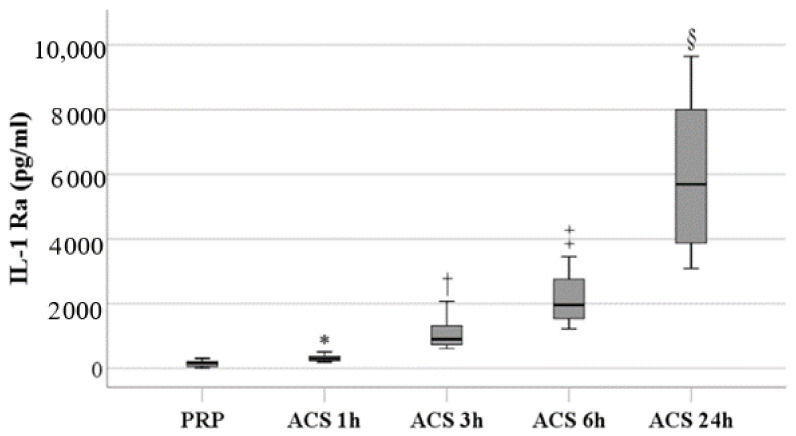
Interleukin-1 receptor antagonist (IL-1Ra) levels of platelet-rich plasma (PRP) and autologous conditioned serum (ACS) with different incubation times. * indicates a significant difference as compared to PRP; † indicates a significant difference as compared to PRP and ACS 1 h; ‡ indicates a significant difference as compared to PRP, and ACS 1 h and 3 h; § indicates a significant difference as compared to PRP and ACS 1 h, 3 h, 6 h.

IL-1Ra levels of ACS preparations for 1 h, 3 h, 6 h, and 24 h were significantly higher than those of PRP, with the medians of 302.0, 902.9, 1963.0, 5689.6 vs. 157.9 pg/mL (*p* < 0.0005). IL-1Ra levels of ACS preparations were significantly increased with an incubation time of up to 24 h. The IL-1Ra level of the ACS preparation for 3 h was significantly higher than that of the ACS preparation for 1 h, with a median of 902.9 vs. 302.0 pg/mL (*p* = 0.0002). Moreover, IL-1Ra levels of ACS preparation for 6 h was significantly higher than those of ACS preparations for 1 h and 3 h, with a median of 1963.0 vs. 302.0 and 902.9 pg/mL (*p* = 0.0002). IL-1Ra levels of ACS preparations for 24 h were significantly higher than those of ACS preparations for 1 h, 3 h, and 6 h, with a median of 5689.6 vs. 302.0, 902.9, and 1963.0 pg/mL (*p* = 0.0002) (Figure 4 and Supplementary Table S1).

#### 3.1.5. TNF-α Levels

**Figure 5:**
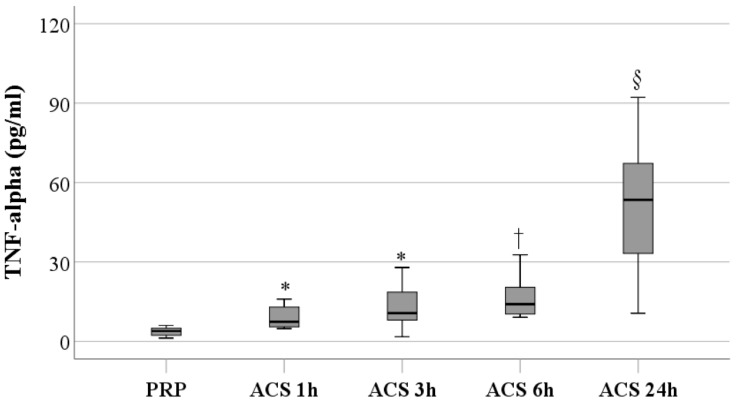
TNF-α levels of PRP and ACS with different incubation times. * indicates a significant difference as compared to PRP; † indicates a significant difference as compared to PRP and ACS 1 h; § indicates a significant difference as compared to PRP and ACS 1 h, 3 h, 6 h.

TNF-α levels of ACS 1 h, 3 h, 6 h, and 24 h were all significantly higher than those of PRP, with the medians of 7.4, 10.64, 14.03, 53.47 vs. 3.88 pg/mL (*p* = 0.0005, 0.0012, 0.0002, and 0.0002), respectively. TNF-α levels within ACS preparations significantly increased with the incubation time; the TNF-α level of ACS at 6 h was significantly higher than that of ACS 1 h, with the medians of 14.03 vs. 7.4 pg/mL (*p* = 0.0002). TNF-α of ACS 24 h was significantly higher than ACS 1 h, 3 h, and 6 h with medians of 53.47 vs. 7.4, 10.64, and 14.03 pg/mL (*p* = 0.0002) (Figure 5 and Supplementary Table S1).

### 3.2. Comparisons of the Efficacy of PRP and ACS Treatment on Pain Relief in Patients with Knee OA

PRP was prepared and injected into the knee after preparation immediately. For ACS treatments, because the higher TNF-α levels appeared in ACS 6 h, and IL-1Ra levels of ACS 3 h were significantly higher than those of PRP, we chose the ACS preparation for 3 h of incubation for the clinical efficacy comparisons between PRP and ACS. The VAS pain scores were evaluated based on patient-reported VAS ratings of 0–10 at baseline and before each treatment.

Sixty patients with Stage III knee OA were enrolled and treated with either PRP or ACS every 2 weeks five times, with 30 patients for each treatment group. The demographic and clinical characteristics are listed in Table 1. There were six (20%) and nine (30%) male patients in the PRP and ACS groups, respectively. The median ages of the two groups were 65.0 and 67.5 years old, respectively. The median BMI was 23.8 and 26.9 for the PRP and ACS groups, respectively. Most patients suffered from OA in both knees and were treated bilaterally, the median pain duration was 24.0 months for patients in the PRP group, and 18.0 months for patients in the ACS group; 36.7% of patients in both treatment groups received oral medicine, and 13.3 % of patients in the PRP group and 33.3% of patients in the ACS group received rehabilitation at baseline. There are no significant differences between the two treatment groups for all the characteristics, except for previous hyaluronic acid treatment.

The outcomes are summarized in Table 2. Eighty percent of patients in the PRP group reported improvement at the second or earlier injection, while 90% of patients in the ACS group reported improvement at the third or later injection. The distribution of the time of patient-reported improvement significantly differs between the two treatment groups (*p* < 0.001). No significant differences between the two treatment groups for the pain VAS at baseline. Patients in the ACS group had more median reduction in VAS pain scores compared to those in the PRP group as −5.0 vs. −4.0 (*p* = 0.028). However, more patients in the ACS group still felt pain (60.0% vs. 20.0%, *p* = 0.003) and needed oral medicine after intra-articular injection (43.3% vs. 10.0%, *p* = 0.007) (Table 2).

## 4. Discussion

In the present study, we tried to find an optimal incubation protocol for ACS preparation to obtain one with lower inflammatory mediators and higher anti-inflammatory factors and growth factors. We did find that ACS 3 h showed lower TNF-α and higher IL-1Ra levels. The FGF-1 and PDGF-BB levels of ACS were markedly increased after incubation for 1 h, and the levels remained at a plateau throughout the 24 h incubation. IGF-1 levels showed a significant increase after incubation for 24 h. IL-1Ra levels increased with the incubation time significantly, while TNF-α showed a turning point for an increase at 6 h, thus incubation for 3 h was selected for clinical evaluation. Meanwhile, except for FGF-1, the levels of IGF-1, PDGF-BB and IL-1Ra in ACS preparations were all significantly higher than those in PRP, suggesting that the clinical efficacy of ACS may be better than that of PRP based on the higher IL-1Ra and PDGF-BB levels in ACS. This postulation was supported by the results of clinical evaluation in patients with knee OA.

The AAOS guideline downgrades the strength of recommendation of PRP from strong to limited due to the mixed results of the studies [4]. One of the possible reasons is the wide range of disease severity among these studies; studies of patients with worse OA showed mixed results [36,37], while those with patients of all stages of OA have favorable results for PRP treatment [37,38]. To minimize the confounder derived from different disease entities of knee OA, we focused on the comparison between PRP and ACS in patients with Stage III knee OA. Results of the present study indicated that ACS has a better ability in pain control compared to PRP because the change of pain VAS from baseline was more in the ACS group compared to that in the PRP group. However, significantly more patients in the ACS group still felt pain and needed oral medicine compared to those in the PRP group at the end of the study, this may be due to more patients with worse pain at baseline choosing ACS administration, even the difference between the two groups did not reach statistical significance, which may relate to the small sample size. A future study with more patients and a more restricted disposition should be conducted to make a solid conclusion. Furthermore, it is notable that patients in the PRP group reported improvement at the second or earlier injection, while most patients in the ACS group reported improvement at the third or later injection. To date, the optimal number of injections of PRP or ACS is still unclear, and no standard recommendation or consensus has been achieved. For PRP, most studies about doses have been conducted with one to three injections and concluded that more injections can provide better and prolonged efficacy. A research and literature review conducted by Subramanyam et al. [39] indicated that three injections yielded superior outcomes to single and double injections. However, one study used up to four to compare with two injections and similarly improved clinical outcomes between the two groups were found [40]. These results may reflect that in most cases, additional doses of PRP may be not necessary, which is consistent with our results. However, this is not the case with ACS. More than three injections may be needed and comparisons of the dose numbers for ACS treatment to clarify the optimal doses have to be performed in the future.

The results of the present study probably cannot be applied to patients with different disease entities and severity levels. Furthermore, there are many risk factors for the progress of knee OA, including sex, age, obesity, and history of trauma. In addition, there are also different risk factors associated with the responses to different treatments. An individualized treatment plan for patients with different characteristics should be developed after more understanding of the pathogenesis of OA and the rationale of individual intervention. Well-controlled studies for patients’ demographic and clinical characteristics are needed for comprehensive planning of ACS preparation and treatment.

Both ACS and PRP are composed of pro-inflammatory and anti-inflammatory factors. Previous studies related to cytokines in knee OA focused on the pathogenesis initially. The investigators noticed that inflammatory factor levels escalated in patients with knee OA [12,13,14]. Developing targeting treatment of the factors, such as synthetic IL-1Ra anakinra or anti-inflammation biologics such as TNF inhibitors is straightforward, and the effects of some biologics have been evaluated and proven in patients with OA [41]. However, the cost of biologics is a barrier to approach. Autologous blood-derived products contain anti-inflammatory factors and are derived from the patient’s own blood, which leads the great safety and minimized cost with therapeutic efficacy. In the present study, we tried to optimize the levels of specific effectors by changing preparation protocols, aiming to be as close as possible to targeting biologics and providing further benefits to patients with keen OA. We chose ACS 3 h for the comparisons based on the balance of the levels of TNF-α and IL-1Ra mainly; other cytokines and growth factors were also considered. The difference in the levels of these effectors in these autologous blood-derived products indicates the impact of different preparation protocols. The results indicated that the selection of the one that has a better efficacy can be conducted based on the levels of specific components in these preparations. The efficacy of ACS and PRP was compatible in the present study. However, the condition we developed in the present study was based on the content analysis of healthy volunteers that may not completely fit the requirement of a treatment since the inflammation status may be different between healthy persons and patients with knee OA, thus the interpretation of the results should be caution with consideration for potential bias. Furthermore, early studies showed that the incubation process of ACS can increase the anti-inflammatory IL-1Ra level while not concomitantly increasing the pro-inflammatory factors, including TNF-α [27,42]; however, our results showed that ACS 24 h contained more IL-1Ra and TNF-α than ACS 1 h. Recent studies showed that in some incubation conditions, concomitant increases of both anti- and pro-inflammatory factors may occur [43,44]. Monocytes are considered to be responsible for the cytokine increase, but the levels of individuals may vary, depending on the size or coated chemicals of the beads that interact with the whole blood [28,45,46]. This is further indicated in that the preparation protocols have impacts on the contents of the products, and materials used for ACS preparation can be evaluated. While the data of contents indicated that PRP had lower levels of IL-1Ra and may limit its ability for pain relief, it further highlights the role of IL-1Ra in the treatment of knee OA. Leone et al. [47] reported that ACS treatment responders had significantly higher levels of IL-1Ra levels than non-responders, with average concentrations of 930 and 200 pg/mL, respectively. This result is consistent with the present one, while the mean IL-1Ra concentration of PRP and ACS 3 h were 157.9 and 902.9 pg/mL, respectively. It is interesting to note that Leone et al. demonstrated that in patients with knee OA who had failed to prior PRP treatment, the response rate of ACS was 67% [47]. This is consistent with the results of the present study that ACS has a better efficacy on pain relief compared to PRP.

Comparisons between ACS and PRP have been evaluated by several investigations; however, more studies are needed for a better solid conclusion because of the controversial results. One study included 96 patients with early knee OA and aimed to compare the function and pain improvements after intra-articular injection of ACS, PRP, hyaluronic acid and steroids. Both blood-derived products had better effects on pain and function, compared to those of hyaluronic acid and steroids, while the percentage changes in pain VAS and the Western Ontario and McMaster Universities (WOMAC) scores from baseline of ACS and PRP did not have any significant difference [48]. The results were different from those of other studies; a randomized clinical trial enrolled 92 patients with Stage II to IV knee OA, and the efficacy of dextrose, PRP, and ACS was compared. The results indicated that dextrose had no substantial change both in pain and function, but ACS and PRP showed improvement after treatment, and ACS was more effective than PRP [49]. Another prospective, controlled, open-label clinical study to compare ACS and PRP directly conducted in patients with Stage II and III knee OA also showed the same results that pain reduction and function improvement presented by WOMAC score of ACS were both better than those of PRP associated with the increases in IL-1Ra levels and synovial fluid viscosity, and the decrease in IL-1b [35]. The reason for the discrepancies among studies is uncertain, since patient inclusion criteria and the preparation protocol of autologous blood products were all different, leading to the difficulty of comparison. Further studies with a larger sample size and comparable baseline characteristics of patients need to be conducted. The component measurements for specific cytokines, chemokines, and growth factors we performed in the present study may provide the opportunity for subjective comparisons, it may be better to include more important cytokines such as IL-10 or IL-35 that play a key role in immunosuppression for a more comprehensive evaluation since the enzyme immune assay is a well-established method for screening several effectors in the same time.

There are some limitations of this study. Pain as the major symptom of OA was assessed by the self-reported VAS changes. This indicator may have a bias because of its subjective nature. However, patient feedback on pain is especially important since it affects the quality of life physically and mentally. Self-reported VAS may also represent the satisfaction level of the treatment they received. However, an objective evaluation tool should be used for comprehensive evaluation. We tried to bridge pre-clinical data and clinical outcomes in the present study; however, bias still exists potentially because of the open-labeled design and relatively small sample size. However, 30 pairs of participants were set for a power of 0.8, an alpha level of 0.05, and an effect size of 0.5 on IL-1Ra and PDGF-BB levels. Based on the assessments of IL-Ra and PDGF-BB levels, the effect size is higher than 0.5 and the study power is over 80% on the significant difference in the anti-inflammatory mediator and growth factors between both groups. Further randomized and double-blinded studies with more participants should be conducted to minimize the possible bias. Growth factors and anti-inflammatory mediators evaluated in healthy volunteers may not truly represent the conditions in patients with knee OA since inflammatory changes are known as the key features and pathogenesis in OA. For more fine adjustment and modification of the autologous blood-derived products for clinical use, content comparisons between healthy persons and patients with knee OA, and patients with different severity should be performed for better understanding to optimize the preparation. Moreover, most patients in both treatment groups were treated bilaterally, which may lead to possible bias when evaluating. However, we decided to treat both sides for the best benefit of the patients. In conclusion, improvement in pain can be reached more with ACS, and the balance of pro-inflammatory factor TNF-α and anti-inflammatory factor IL-1Ra of the autologous blood-derived products should be considered in the clinical practice of knee OA management.

## Figures and Tables

**Table 1:** Demographic and clinical characteristics in patients with Stage III osteoarthritis of the knee.

	PRP (*n* = 30)	ACS (*n* = 30)	*p*-Value
Sex, male	6 (20.0%)	9 (30.0%)	0.552
Age (year)	65.0 (60.0, 70.0)	67.5 (59.0, 71.0)	0.554
BMI (kg/m^2^)	23.8 (21.7, 27.8)	26.9 (24.0, 28.5)	0.064
Treatment side			0.052
Bilateral	30 (100.0%)	25 (83.3%)	
Left	0 (0.0%)	4 (13.3%)	
Right	0 (0.0%)	1 (3.3%)	
Pain duration (month)	24.0 (12.0, 36.0)	18.0 (12.0, 30.0)	0.099
Previous treatment *			
Tropical medicine	0 (0.0%)	2 (6.7%)	0.492
Oral medicine	5 (16.7%)	8 (26.7%)	0.532
Rehibition	2 (6.7%)	2 (6.7%)	>0.999
Physical therapy	0 (0.0%)	1 (3.3%)	>0.999
Manual Therapy	1 (3.3%)	0 (0.0%)	>0.999
Acupuncture	4 (13.3%)	1 (3.3%)	0.353
Acupotomy	1 (3.3%)	0 (0.0%)	>0.999
Hyaluronic acid	2 (6.7%)	10 (33.3%)	0.021 ^†^
Glucose	0 (0.0%)	1 (3.3%)	>0.999
PRP	1 (3.3%)	1 (3.3%)	>0.999
Nutritional supplements	4 (13.3%)	1 (3.3%)	0.353
None	13 (43.3%)	8 (26.7%)	0.279
Treatment at baseline			
Oral medicine	11 (36.7%)	11 (36.7%)	>0.999
Rehabilitation	4 (13.3%)	10 (33.3%)	0.125

Data are expressed by count and percentage, except for age, BMI, and pain duration are presented by median and interquartile range. * Patients may receive several kinds of treatments. ^†^ Indicates a significant difference observed between groups.

**Table 2:** Outcomes of PRP or ACS treatment in patients with Stage III osteoarthritis of the knee.

	PRP (*n* = 30)	ACS (*n* = 30)	*p*-Value
Pain VAS at baseline	6.0 (4.0, 8.0)	7.0 (6.0, 8.0)	0.098
Pain VAS at the end of the study	1.0 (1.0, 2.0)	1.0 (0.0, 2.0)	0.822
Change of pain VAS from baseline	−4.0 (−6.0, −2.0)	−5.0 (−7.0, −4.0)	0.028 ^†^
After which injection the patients reported improvement			<0.001 ^†^
1st	14 (46.7%)	2 (6.7%)	
2nd	10 (33.3%)	1 (3.3%)	
3rd	5 (16.7%)	13 (43.3%)	
4th	0 (0.0%)	4 (13.3%)	
5th	1 (3.3%)	10 (33.3%)	
Still feel pain at the end of the study	6 (20.0%)	18 (60.0%)	0.003 ^†^
Still need oral medicine at the end of the study	3 (10.0%)	13 (43.3%)	0.007 ^†^

Pain VAS at baseline, pain VAS at the end of the study, and change of pain VAS from baseline are presented by median and interquartile range. The other data are expressed by count and percentage. ^†^ Indicates a significant difference observed between groups.

## Data Availability

Data will be made available on request.

## Supplementary Materials

The following supporting information can be downloaded at: https://www.mdpi.com/article/10.3390/biom13030555/s1, Table S1: Cytokine levels of PRP and ACS.
