# Supplementary material for: Comparisons of Cytokines, Growth Factors and Clinical Efficacy between Platelet-Rich Plasma and Autologous Conditioned Serum for Knee Osteoarthritis Management
Source: Biomolecules. 2023 Mar 17;13(3):555. doi: 10.3390/biom13030555 (PMC10046072; doi:10.3390/biom13030555)
Supplement: Supplementary file 1 [file biomolecules-13-00555-s001.zip › biomolecules-2124041-supplementary.pdf]

biomolecules

MDPI

Article

Comparisons of Cytokines, Growth Factors and Clinical Efficacy between Platelet-Rich Plasma and Autologous Conditioned Serum for Knee Osteoarthritis Management

Pen-Gang Cheng [1], Kuender D. Yang [2], Liang-Gie Huang [3], Chi-Hui Wang [4] and Wang-Sheng Ko [5,*]

[1] Department of Orthopaedics, Fu-Ya Medical Clinic, Taichung 40764, Taiwan
[2] Department of Medical Research, Mackay Children's Hospital, Taipei 10449, Taiwan;
[3] Department of Medical Research, Mackay Memorial Hospital, Taipei 10449, Taiwan
[4] Department of Stomatology, Taichung Veterans General Hospital, Taichung 40705, Taiwan
[5] Department of Orthopaedics, Cheng-Ching General Hospital, Taichung 40764, Taiwan
[6] Department of Internal Medicine, Kuang-Tien General Hospital, Taichung 43302, Taiwan
* Correspondence: ker200448@yahoo.com.tw; Tel: +886-1915251829; Fax: +886-422510800

Supplementary Materials

Table S1. Cytokine levels of PRP and ACS.

	PRP (*n* = 13)	ACS 1 h (*n* = 13)	ACS 3 h (*n* = 13)	ACS 6 h (*n* = 13)	ACS 24 h (*n* = 13)	*p*-Value
FGF-1 (pg/mL)	110.7 (92.2, 128.3)	115.1 (107.4, 126.4)	101.9 (97.5, 126.4)	101.9 (95.4, 144.0)	99.9 (95.4, 110.7)	0.099
PDGF-BB (pg/mL)	2035.2 (1020.4, 2652.8)	11414.8 (7970.5, 12979.7) [a]	9512.7 (8612.1, 12112.8) [a]	10001.2 (7830.4, 11206.5) [a]	10382.5 (7893.8, 13500.9) [a]	<0.001 *
IGF-1 (pg/mL)	43790.8 (36243.5, 54580.2)	63019.8 (46079.0, 69453.9) [a]	64724.5 (47443.9, 82279.1) [a]	62477.9 (51201.5, 77844.3) [a]	72194.0 (63309.0, 76162.0) [a,b]	<0.001 *
IL-1 Ra (pg/mL)	157.9 (55.4, 214.3)	302.0 (248.1, 382.3) [a]	902.9 (731.8, 1318.4) [a,b]	1963.0 (1537.0, 2753.9) [a,b,c]	5689.6 (3873.4, 7996.2) [a,b,c,d]	<0.001 *
TNF-α (pg/mL)	3.88 (2.28, 4.95)	7.40 (5.48, 12.96) [a]	10.64 (8.04, 18.56) [a]	14.03 (10.31, 20.43) [a,b]	53.47 (33.23, 67.22) [a,b,c,d]	<0.001 *

**p*-Value < 0.05 in the corresponding Friedman's test, which implies at least a significant difference exists between every two conditions of PRP, ACS 1 h, ACS 3 h, ACS 6 h, and ACS 24 h. [a] Indicates a significant difference as compared to PRP; [b] Indicates a significant difference as compared to ACS 1 h; [c] Indicates a significant difference as compared to ACS 3 h; [d] Indicates a significant difference as compared to ACS 6 h.
